# Disrupted Sense of Agency as a State Marker of First-Episode Schizophrenia: A Large-Scale Follow-Up Study

**DOI:** 10.3389/fpsyt.2020.570570

**Published:** 2020-12-18

**Authors:** Eva Kozáková, Eduard Bakštein, Ondřej Havlíček, Ondřej Bečev, Pavel Knytl, Yuliya Zaytseva, Filip Španiel

**Affiliations:** ^1^Department of Applied Neuroscience and Neuroimaging, National Institute of Mental Health, Klecany, Czechia; ^2^Department of Psychology, Faculty of Arts, Charles University, Prague, Czechia; ^3^Department of Cybernetics, Czech Technical University in Prague, Prague, Czechia; ^4^Department of Neurology, St. Anne's University Hospital and Faculty of Medicine, Masaryk University, Brno, Czechia; ^5^Department of Psychiatry and Medical Psychology, Third Faculty of Medicine, Charles University, Prague, Czechia; ^6^Human Science Center, Institute of Medical Psychology, Ludwig Maximilian University, Munich, Germany

**Keywords:** schizophrenia, sense of agency, self-disturbances, positive and negative symptoms, follow-up

## Abstract

**Background:** Schizophrenia is often characterized by a general disruption of self-processing and self-demarcation. Previous studies have shown that self-monitoring and sense of agency (SoA, i.e., the ability to recognize one's own actions correctly) are altered in schizophrenia patients. However, research findings are inconclusive in regards to how SoA alterations are linked to clinical symptoms and their severity, or cognitive factors.

**Methods:** In a longitudinal study, we examined 161 first-episode schizophrenia patients and 154 controls with a continuous-report SoA task and a control task testing general cognitive/sensorimotor processes. Clinical symptoms were assessed with the Positive and Negative Syndrome Scale (PANSS).

**Results:** In comparison to controls, patients performed worse in terms of recognition of self-produced movements even when controlling for confounding factors. Patients' SoA score correlated with the severity of PANSS-derived “Disorganized” symptoms and with a priori defined symptoms related to self-disturbances. In the follow-up, the changes in the two subscales were significantly associated with the change in SoA performance.

**Conclusion:** We corroborated previous findings of altered SoA already in the early stage of schizophrenia. Decreased ability to recognize self-produced actions was associated with the severity of symptoms in two complementary domains: self-disturbances and disorganization. While the involvement of the former might indicate impairment in self-monitoring, the latter suggests the role of higher cognitive processes such as information updating or cognitive flexibility. The SoA alterations in schizophrenia are associated, at least partially, with the intensity of respective symptoms in a state-dependent manner.

## Introduction

Disturbances of the self have been considered as a core unifying marker of schizophrenia during the early days of the genesis of this diagnostic category (1) but also in recent accounts (2, 3). The most prominent symptoms of schizophrenia, first-rank symptoms, are related to the impairment of sense of agency [SoA, i.e., the subjective experience of being in control of one's own actions; (4, 5)]. Alterations in the ability to experience self/other boundaries coherently and reliably have been documented simultaneously in phenomenologically oriented (6, 7) and also in behavioral studies (8). The relation between a global experience of oneself as an acting agent (or its alterations, as seen in schizophrenia symptoms), and the experience of agency in specific model situations (i.e., SoA tasks using manipulation of sensory feedback) seems intricate. In his seminal work, Frith (9) proposed that the underlying mechanism of schizophrenia symptoms (especially first-rank symptoms) lies in self-monitoring impairment, where expectations of the sensory outcome of one's actions don't fit the actual incoming sensory signals. Linking clinical symptoms with performances in SoA experiments yields heterogeneous results with studies suggesting the importance of both positive (10) and negative symptoms (11). Longitudinal studies exploring the connection between symptoms' presence and performance in SoA tasks in time may shed more light on the nature of this relationship. Change in SoA performance associated with changes in symptoms severity would indicate their interconnectedness and a state-dependent characteristic of SoA in schizophrenia. A possible lack of such association would suggest the notion that SoA alterations are a trait characteristic of schizophrenia, a possible endophenotype, i.e., heritable biomarker (12). Endophenotypes used as quantitative measures help to connect observable behavioral level (e.g., performance in working memory tasks) with the genetic substrate, and are, thus, considered useful in enriching our understanding of the pathway from genes to functional alterations in schizophrenia (13).

Phenomenological analyses of self-disturbances (SDs) describe impairments in the first-person perspective, sense of coherence, and self/other confusion (14). SDs are also present in its broader spectrum, including schizotypal personality disorder or non-psychotic family members of schizophrenia patients, and their presence in healthy individuals might indicate elevated risk for future development of the illness (15). In line with phenomenologically oriented approaches, many studies in schizophrenia report aberrances in self-experiences on a behavioral level, especially in various forms of bodily/motor representations [for review see meta-analysis (8)], including studies of SoA using manipulation of visual feedback of one's own actions (16–20). The most consistent conclusion from these studies is that in schizophrenia the precision of bodily/motor representations seems weaker, which makes false inferences about causal agents more likely.

Despite numerous attempts, application of clinical scales [e.g., Positive and Negative Symptom Scale, PANSS; (21)] has not helped to distill unequivocally a cluster of symptoms that would correspond to behavioral performance in SoA experiments (22, 23). The majority of findings generally pinpoint the significant role of positive symptoms in alterations of SoA, which is, in most cases, linked with exaggerated self-agency [i.e., attribution of causation of events to self; (8, 11, 24)], although diminished self-agency has also been documented (23, 25). While finding alterations of SoA in schizophrenia patients compared to controls, no significant correlations have been found between performance in SoA tasks based on explicit judgments of agency and either PANSS total or subscale scores (26), or passivity symptoms (25). A significant limitation of the reviewed studies is small sample sizes of the target patient groups.

Regarding the discussion about state vs. trait nature of self-disturbances in schizophrenia (i.e., present only in acute phases, or not), phenomenologically oriented studies support the trait-like character of SDs (3, 6, 27, 28). In behavioral studies, evidence can be found for both sides; there are findings of alteration of self-monitoring as a trait (10, 25, 26), state (29), or intermediate-marker (30, 31).

## Current Study—Aims and Hypotheses

The main aim of this study was to examine the relationship between schizophrenia symptomatology and SoA in a large sample of first-episode schizophrenia (FES) patients, in which there are no effects of long-term medication or hospitalization as in chronic patients. (1) We expected to corroborate previous findings of altered SoA in schizophrenia. Next, (2) we expected that such a specific self-attributional deficit would be detectable even after controlling for possible cognitive confounding factors (e.g., attention, motor speed) that may otherwise affect the performance during the motor task in patients. We directly addressed this potential confounding effect by adding a control task. (3) Since the deficit in self-monitoring is supposedly the underlying mechanism of the first-rank symptoms, we hypothesized that altered self-agency would be linked to (3a) positive symptomatology captured by PANSS Positive factor (32) and (3b) especially to symptoms related to self-disturbances. Finally, (4) we focused on the stability of the relationship between the symptomatic (PANSS) and behavioral (SoA score) domains. We tested whether a change of symptoms over time would lead to a consistent change of SoA performance, indicating its state-dependent nature.

## Methods

### Participants

Participants were recruited into the study upon participation in the Early-Stage Schizophrenia Outcome (ESO) Study (33), which examines longitudinal changes in schizophrenia including neurocognitive, behavioral, genome-wide sequencing measures in relation to clinical outcomes. Of 246 first-episode schizophrenia (FES) patients and 181 healthy controls (HC) included in this follow-up study, 161 FES (90 males, i.e., 55.9%) and 154 HC (69 males, i.e., 44.8%) were able to perform the tasks according to instructions and were included in the data analyses (Supplementary Table 1; see participants' characteristics in Table 1; the criteria are described in Supplementary Material 2). Of the included participants, 82 patients and 65 controls had complete data from both tasks during a visit (taken visit 1 and 2 together). Patients were recruited into the study during their hospitalization in some of Czech inpatient psychiatric facilities participating in the ESO Enrolment Network run by the National Institute of Mental Health. Only patients with the diagnosis of schizophrenia spectrum disorder according to ICD-10 that was confirmed at the time of follow-up were included in analyses (i.e., F20, F23, F25; *N* = 97, 55, 9; respectively). The inclusion criteria for the participants were an age between 18 and 60 years and an absence of organic brain disorders or serious neurological illness. All patients were medicated at the time of participation. Healthy controls, additionally, were required neither to have a history of psychiatric disorders themselves nor in their close family (i.e., first-degree family members). The healthy control subjects were recruited via an advertisement from a similar socio-demographic background as FES participants. Participants were assessed twice—at Visit 1 (baseline; in patients referring to the time of admission to a psychiatric hospital) and at Visit 2 (follow-up after ~1 year). The study was approved by the Ethics committees of the institutions involved. Written informed consent was obtained from all participants before the assessments.

**Table 1:** Characteristics of participants.

	**Patients**		**Controls**	
	**Visit 1**	**Visit 2**	**Visit 1**	**Visit 2**
*N*	117	87	117	98
Age	29.1 (7.5)	30.9 (7.3)	27.7 (6.5)	30.2 (6.8)
Age at onset	28.5 (7.63)	–	–	
Sex	71 M / 46 F	42 M / 45 F	50 M / 67 F	50 M / 48 F
Duration of illness (months)	7.4 (8.9)	22.6 (10.7)	–	–
Neuroleptic dosage (CPZ equiv.)	361 (218)	191 (172)	–	–
PANSS Total	57.2 (14.8)	48.1 (11.9)	–	–
**PANSS Factors**				
Positive	7.5 (3.1)	5.6 (3.0)	–	–
Negative	13.5 (5.0)	11.6 (4.3)	–	–
Disorganized	5.8 (2.1)	5.2 (1.7)	–	–
Excited	5.2 (1.7)	4.93 (1.5)	–	–
Depressed	5.9 (2.3)	4.9 (1.9)	–	–
Self-cluster	12.8 (2.6)	11.2 (1.8)	–	–

*Mean (SD). N, number of participants; M, males; F, females; CPZ equiv., chlorpromazine equivalent; GAF, Global Assessment of Functioning Scale; PANSS factors according to Wallwork et al. (32); Self-cluster: a priori defined self-related items*.

## Measures

### Sense of Agency Task: Self-Monitoring Motor Task

We used a motor task with a continuous report of experienced agency over a presented movement with a varying degree of distortion, the same as in Spaniel et al. (34) (see Supplementary Material 1). Using a joystick game controller device to move a cursor projected on a screen, participants were instructed that, from time to time, the experimenter would be influencing the cursor's movement (i.e., Other-condition), while at other occasions the cursor would follow the movement of their joystick solely (i.e., Self-condition). During the Self-condition, they were supposed to move the cursor outside the central area, while in the Other-condition, they were supposed to keep it inside.

In fact, the displayed movement was periodically distorted by adding distortion to the participants' motion according to a predefined sequence of distances and angles, which was the same for all participants (Supplementary Material 1, Supplementary Figure 1B). The task sequence consisted of 12 Self and 12 Other blocks, presented in alternating order (Self-Other-Self-...). Based on debriefings after the task, none of the participants noticed the regular pattern of the Self/Other condition changes.

The SoA performance (“SELF-score”) was calculated as a percentage of time spent in the peripheral area during the Self-condition (i.e., a period without visual feedback distortion) that, according to the prior instructions reflected patients' subjective experience of agency. This way, the “SELF-score” reflected the accuracy of participants' self-agency judgments. We decided not to include the performance from the Other-condition because in that condition, we cannot separate clearly whether the movement in either area of the square was due to participants' decision or due to the distortion. Nevertheless, to gain further insight into participants' performance, we performed an exploratory analysis of the data from both conditions using a Signal detection theory approach (SDT; Supplementary Material 4).

### Color Task: Sensorimotor Control Task

The control task (referred to as “Color task”) is a modification of the SoA task; there is no movement distortion, and the instruction to move the cursor outside or inside the square is based on the cursor's color which changes between green and red irregularly. The task lasts 1 min; the interval of color changes is between 3 and 7 s, with 12 changes in total. This motor task was designed to measure the subjects' general cognitive capacities such as attention, motor precision and response. The performance score in the Color task (i.e., COLOR-score) was calculated as a percentage of time spent in the correct corridors.

### Tasks Procedure

Before the main experiment, participants were instructed about the principles of both tasks, after which they practiced these tasks using a tablet touchscreen to control the cursor that was connected to a monitor for a presentation of the cursor movement. The experimenters made sure the participants understood the principle of the tasks before starting the experiments. During each visit, participants performed the SoA (8 min) and Color (1 min) tasks inside the MRI scanner using a joystick to control the presented cursor.

Of note, the sense of agency task was introduced into the long-term ESO study later on via an amendment to the study protocol. Some participants have therefore experienced the SoA task for the first time at Visit 2. This was taken into account during the data analysis.

The fMRI results coming from the same study were published in Spaniel et al. (34). In the study, the neuronal activation during the SoA task overlapped with the areas previously highlighted to be specific for self-related processing (35).

### Clinical Symptoms Assessment

At each visit, patients were assessed by trained clinicians with the Positive and Negative Symptom Scale [PANSS; (21)], widely used in clinical research. First, we used the categorization of PANSS based on a meta-analysis of factor-analyses into positive, negative, disorganized, excited, and depressed factors (32). Next, to further refine the focus on items arguably connected to self-disturbances, we created an additional subscale (so-called Self-cluster) prior to data analyses based on the Positive PANSS subscale with the omission of items that did not seem relevant to self-processing (i.e., P2-conceptual disorganization, P4-excitement, and P7-hostility. The included items are P1-delusions, P3-hallucinatory behavior, P5-grandiosity (inversely scored), P6-suspiciousness/persecution. While P1 and P3 are linked to self-attributional alterations, P5 and P6 indicate a bias or experiencing agency (36–38).

### Statistical Analyses

The resulting trajectories of the joystick movements were processed and scores calculated in Matlab (39). Statistical evaluations and models were calculated using R (40).

For between-group comparisons, the student's *t*-test or the non-parametric Wilcoxon's rank-sum test were used depending on prior assumptions and visual inspection of normality. Where the analyses included repeated measurements due to the longitudinal design (i.e., the models of the association between task scores and group and the model of task scores and PANSS), we used linear mixed-effect models (LME), which model individual participant's effects (random effects), in addition to the general trend—the fixed effects. Statistical inference from the LME's was performed using likelihood ratio tests on hierarchical model structures using ANOVA. The significance level considered was alpha = 0.05. The results were corrected for multiple comparisons using the Holm-Bonferroni method for the familywise error rate, where multiple comparable exploratory models were assessed—as indicated in the results section. The analysis of associations between longitudinal differences in task and PANSS scores was performed using linear regression. As the participants were not paired regarding sex nor age, and these characteristics might have an effect on the performance in the tasks, we controlled for these variables in the analyses.

## Results

### Overview of Performance Scores

**Figure 1:**
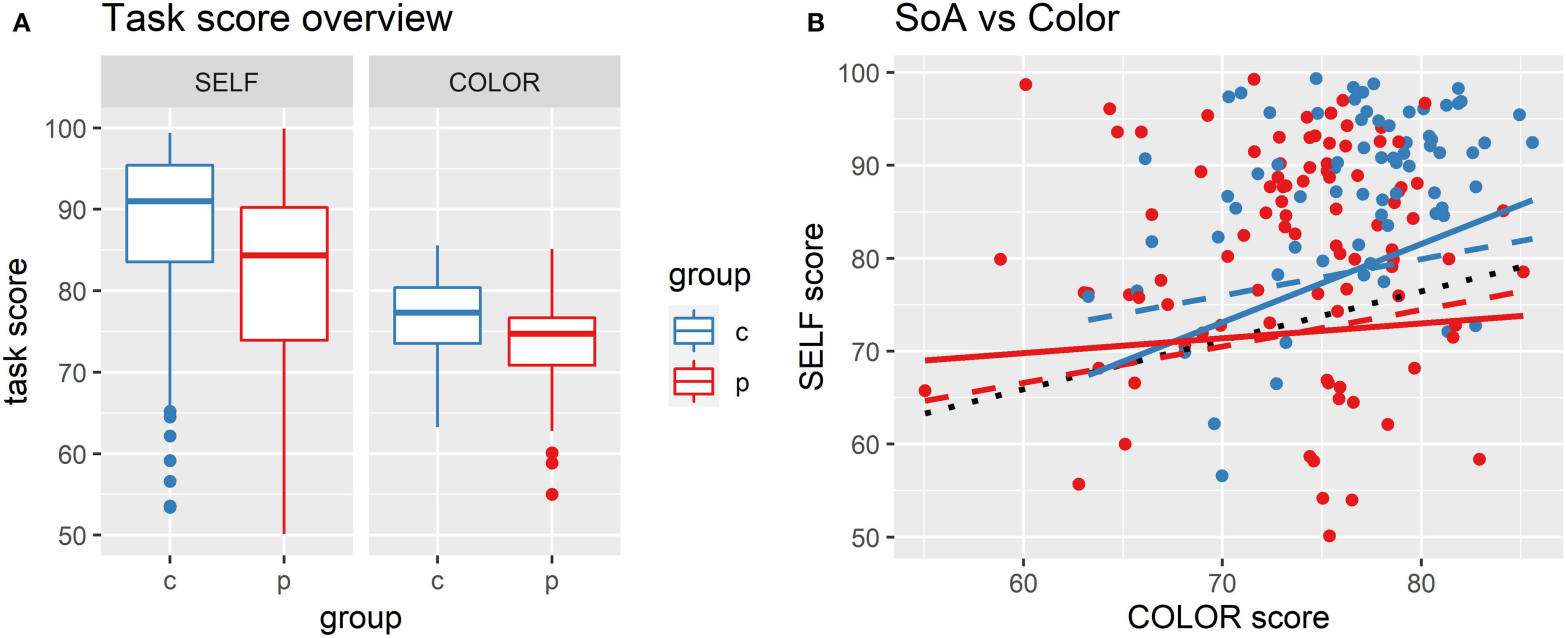
Performance in the sense of agency and the color tasks. **(A)** Distribution of scores in SoA and Control task: Overview of the distribution of scores, showing higher scores achieved by the control group in both the SoA and Control task. c, healthy controls; p, patients. **(B)** Comparison of three general mixed-effect models (GLM) using ANOVA: Baseline model (A—black dotted line) assumes that SELF-score can be fully explained by the COLOR-score (performance in the control task). Model B (dashed red and blue) assumes that FES and HC differ in intercept with patients scoring significantly worse in the SoA task, but the slope is identical. The model C (solid red and blue) additionally assumes a different slope for each group. This last model explained the results better than the previous simpler models.

The groups differed in both the SoA and the Color tasks, with the healthy controls scoring significantly higher in both tasks in all comparisons—see Table 2 and Figure 1A. There was an apparent improvement from Visit 1 to Visit 2 in both groups, especially in the SELF-score (see Table 2; Supplementary Figure 2). The possible reason for that can be attributed mainly to the two following causes: (i) familiarity with the task, and (ii) the improvement in the clinical state of the patient group.

**Table 2:** Score in the SELF and COLOR tasks.

**VISIT**	**TASK**	**Patients**	**Controls**	**Difference between Patients and controls**
V1	Self	80.2 (12.2, *N* = 117)	86.4 (10, *N* = 117)	<0.001, *W* = 9,037, *d* = 0.56
	Color	70.5 (11.2, *N* = 48)	74.8 (9.12, *N* = 33)	0.011, *W* = 1,057, *d* = 0.63
V2	Self	82.8 (12.1, *N* = 87)	90.4 (8.22, *N* = 98)	<0.001, *W* = 5,890, *d* = 0.74
	Color	73.7 (6.0, *N* = 44)	76.5 (4.5, *N* = 39)	0.018, *W* = 1,118, *d* = 0.53
V1+V2	Self	80.6 (11.9, *N* = 161)	87.6 (9.2, *N* = 154)	<0.001, *W* = 29,677, *d* = 0.63
	Color	73.5 (5.8, *N* = 82)	76.6 (4.8, *N* = 65)	<0.001, *W* = 4,433, *d* = 0.58

*Mean (SD, N) mean values and standard deviations for all tasks and visits, N, number of participants; Self, SELF-score performance in SoA task; Color, COLOR-score, i.e., performance in the control Color task; Difference between both groups: p-uncorrected, Wilcoxon test, Cohen's d effect sizes*.

To evaluate the between-group difference in SELF-score, we accounted for the performance in the Color task, repeated measurements, familiarity effect, and the difference between visits in the following analysis.

### SoA Task Controlled for Basic Sensorimotor Performance

We compared three linear mixed-effect models using ANOVA to test the best model fit in order to explore the difference between patient and control group in the SoA task performance (SELF-score) and its relation to sensorimotor/cognitive functioning (COLOR-score; Figure 1B; Supplementary Table 2). In all models, the dependent variable was the SELF-score; the independent variables included the COLOR-score, sex, and age. The random effects included a random intercept for each patient.

The baseline model (*a*) assumed that the performance in the SoA-task reflected only basic sensorimotor and cognitive capacities, i.e., across all participants, the SELF-score would depend solely on the COLOR-score. The model additionally controlled for sex, age at the first visit, and the number of visits. No between-group differences were considered here.

The second model (extended model, *b*) assumed, in addition, that there is a constant difference between the groups, i.e., both groups have the same slope but different intercept. (i.e., patients differ from controls in the SELF-score, but this difference is unrelated to capacities controlled for by the Color task). Comparison of the baseline (*a*) and the extended model (*b*) showed a significantly better fit of the model (*b*) to the data, ANOVA, *p* = 0.003.

The third model (*c*) included the interaction of the slope with the group (i.e., a different degree of dependence between the COLOR- and SELF-scores in each group). A direct comparison of this model (*c*) to the model (*b*) yielded a significantly better fit to the data, ANOVA, *p* = 0.029. A possible familiarity effect between visits was tested by the additional model (*d*), which did not bring significant improvement in the likelihood test, ANOVA, *p* = 0.635; Supplementary Tables 2, 3.

To evaluate the relationship between the COLOR and SELF scores in each group separately, we evaluated the model *(a)* separately on patient data, model *(ap)*, and on data of healthy controls, model *(ac)*. The models showed a significant association between the two scores on the patient group, β = 0.85, *p* < 0.001, but no significant association on the patient group, β = 0.16, *p* = 0.469.

### SoA and Control Task Performance in Connection to Concurrent Symptoms Presence (PANSS) in Patients

**Figure 2:**
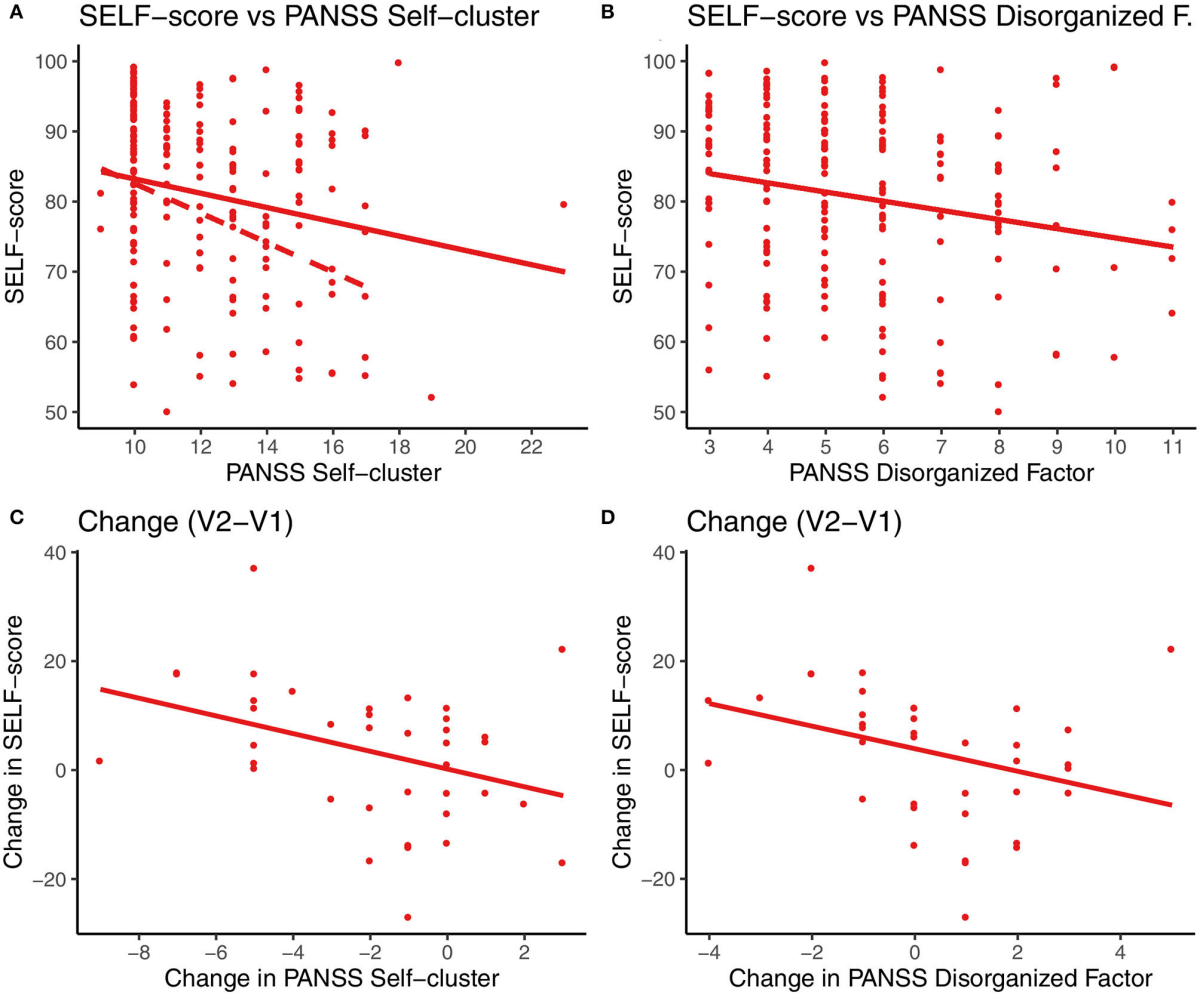
Dependence of SELF-score on PANSS self-cluster and disorganized factor. Top: the SELF-score showed significant association with selected PANSS subscales: **(A)** the a-priori defined PANSS Self-cluster and **(B)** Disorganized factor (solid red line, mixed-effect model). In panel **(A)** removal of the outliers from the model (Self-cluster value>20—red empty circle) showed even stronger association (red dashed line, β = −1.12 *p*_corr_ = 0.020). Bottom: **(C)** the changes in symptoms severity between Visit 1 and 2 (i.e., V1 and V2) of Self-cluster and **(D)** of the Disorganized factor that both showed a significant linear association with the SELF-score.

We found a statistically significant negative correlation between the SELF-score and the severity of the a priori selected Self-cluster symptoms, β = −1.02, *p*_corr_ = 0.029 (Figure 2A), and the Disorganized factor, β = −1.31, *p*_corr_ = 0.017 (Figure 2B), as evaluated by a linear mixed-effects model with random intercepts for each participant using the data from both visits. Other factors (Positive, Negative, Excited, and Depressed) were non-significant after Holm-Bonferroni multiple comparisons correction. We found no significant association between the performance in the Color task and any of the subscales (Supplementary Table 5).

### Longitudinal Changes

To further investigate the relationship between the SoA task and symptomatology with respect to the longitudinal changes in patients' state, we evaluated between-visit differences in the SELF-score and PANSS disorganized factor and the Self-cluster (scores in Visit 2—Visit 1). The association between the SELF-score was significant for the PANSS Self-cluster changes, *R*^2^ = 0.13, *F*_(1, 36)_ = 5.47, *p* = 0.025 (Figure 2C), as well as for the Disorganized factor, *R*^2^ = 0.11, *F*_(1, 36)_ = 4.26, *p* = 0.046 (Figure 2D).

## Discussion

In this follow-up study, we explored the links between alterations in SoA, basic sensorimotor capacities, and phenomenological expressions (i.e., clinical symptoms) in first-episode schizophrenia. We used continuous-report self-attribution (SoA) and sensorimotor control (Color) tasks, and, in patients, PANSS to assess clinical symptoms. The results of our study are consistent with the previous reports on the impaired self-attribution of agency in schizophrenia patients (8, 25, 26, 41). In line with previous findings (42, 43), patients also showed an impairment in a broader cognitive/sensorimotor domain reflected in the Color task performance. This can be attributed to factors such as psychomotor retardation linked to medication, attention, and motivation deficits. Nonetheless, the difference in SoA between schizophrenia patients and healthy individuals could not be explained merely by patients' lower sensorimotor performance even when possible influences of age or sex were controlled for. Likely, SoA impairment goes beyond changes in basic sensorimotor processes. Another key finding in our study is that the impaired self-attribution of agency was linked to the current severity of symptoms reflecting both self-disturbances and disorganization.

### Performance in the SoA and Color Tasks

In our SoA task, patients were less capable of recognizing their actions, i.e., periods of time when the presented movements were entirely under their control and not influenced by distortions. The result of altered SoA in schizophrenia is in line with several other experimental findings (8). Moreover, not only patients performed worse than healthy individuals in SoA and control tasks, both patients and healthy controls also differed in how strongly their performances in both tasks were related. While in healthy individuals, the performance in the SoA and Color tasks were strongly correlated, we found no such association in patients. In essence, we could assume that the performance of the healthy subjects—with supposedly intact self-monitoring—in the SoA task is limited by basic sensorimotor capacities. On the other hand, in patients, performance in the SoA task could not be explained by basic sensorimotor abilities to the same degree as in healthy subjects, indicating a disruption in additional processes. Previously it has been suggested that comparator or cue-integrative mechanisms are the basis for SoA (44, 45).

Because of the nature of the SoA task (uncertainty about intended responses during the Other-condition due to the presence of motion distortion), we analyzed primarily only the Self-score from the Self-condition, i.e., the ability to recognize self-generated movements. This “true positive rate” was decreased in the patient group compared to controls, in line with some other studies (18, 23). Nevertheless, we have performed an exploratory SDT analysis on both conditions (Supplementary Material 4), which suggests that the decreased true positive rate was more likely due to generally lower sensitivity to discriminate the cause of the actions rather than a bias toward under-attribution of agency. However, these results should be taken with caution (Supplementary Material 4). In any case, neither of these results indicates an over-attribution of agency to oneself in schizophrenia as the majority of studies using explicit SoA tasks reports do (8).

While there is a general consensus about SoA alterations in schizophrenia, the specific details regarding the elemental parts (e.g., sensitivity, bias) somewhat vary (8). The disunity of results might be caused by the methods used in the SoA field. Although they are often inspired by the model of self-monitoring mechanisms, their concrete implementation varies greatly (8, 11, 25, 26, 41, 46). One possible way to explain and synthesize our findings with those from other studies is that, in schizophrenia, there might be a general tendency for excessive SoA in behavioral tasks that employ discrete trials (16–18, 22). It's been suggested that over-attribution of agency to oneself is a compensatory strategy for patients to regain a sense of control in ambiguous situations (16–19). In this type of paradigms, agency estimation is performed trial-by-trial. In our continuous-report task, the Self-condition always came after the Other-condition. Thus, the patients' worse performance in recognition of their own agency signifies an affected ability to realize regaining of control that comes after a period of considerably distorted feedback of their actions. A prominent theory suggests that the underlying mechanisms behind SoA can be explained by the comparator model (9), where expected and real sensory information of one's own actions tag these actions as self-produced or not. This theory was further complemented by the optimal-cue integration/ multifactorial-weighting model, which “specifies different agency cues on different levels that are weighted according to their relevance in the specific context” [(45), p. 57]. Thus, in our task, not only the internal predictions about the consequences of one's movement are compared with the actual sensory feedback, but also the preceding experience of lack of agency may come into play as an additional cue. This way, a possible tendency to self-attribute extensively might compete with the expectations of the presence of intrusions into own actions from the preceding context. Impaired self-monitoring might thus obscure the updating of available information and hinder the ability to notice the absence of discrepancy between sensory expectations and real sensations. Indeed, a study similar to ours (23) using a continuous-report task found longer durations of false-negative agency judgments in schizophrenia, which was associated with cognitive impairment. Thus, the decreased patients' ability to recognize own movements we observed might be caused by the two processes—by weaker bodily/motor representations (self-monitoring) and impaired cognitive ability to update information (flexibility).

### SoA and Symptomatology

In the present study, SoA impairment was associated with the severity of the positive symptoms from the defined Self-cluster of PANSS and also with the severity of the symptoms within the Disorganized Factor based on the five-factor PANSS structure (32). The stronger the severity of these symptoms was, the lower the ability to self-attribute own actions was observed. The link between SoA and positive symptoms, mostly delusions and hallucinations was confirmed in various studies (10, 16, 18, 19, 26, 47), albeit also opposing results have been reported (22, 23). Indeed, as initially proposed by Schneider (48), first-rank symptoms are deeply rooted in the disorder of the self. In acute psychosis, the boundaries between “self and other” are violated and one's own actions and thoughts are ascribed to the external agents or other's actions are interpreted as one's own. On the experimental level, Nordgaard et al. (49) confirmed that the increased level of self-disturbances was related to the probability of reporting of the first-rank symptoms.

Studies have demonstrated that the Disorganized factor containing conceptual disorganization, difficulty in abstraction, and poor attention was associated with neurocognitive deficits in schizophrenia (50), in particular with executive functions (51). Our Color task arguably controls for attentional processes to a large degree. Since the control task score was not correlated to the Disorganized factor, probably conceptual disorganization and/or difficulty in abstraction drive more the relationship between SoA impairment and severity of disorganized symptoms.

### SoA Alterations in Follow-Up

In patients who underwent follow-up testing, we focused on the factors that showed a significant relationship with the ability to recognize their own agency correctly (i.e., the Self-cluster and the Disorganized factor). The change in SoA ability was strongly associated with changes in both factors, suggesting it is state-dependent. Such a result is in contrast to experimental evidence from cross-sectional studies comparing acute and stable patients suggests the severity of current symptoms does not play a crucial role in SoA alterations (10, 25, 47). Nonetheless, our results do not rule out the possibility of SoA impairment being, at the same time, a trait characteristic of schizophrenia regardless of the current severity of the symptoms. In this line, a possibility of an “intermediate trait vulnerability marker” in source-monitoring studies was proposed, where both state- and trait-like quality of SoA alterations can be present simultaneously (30, 52).

### Limitations

There are several limitations of our study. First, the SoA task was introduced into the already running long-term ESO study, which has several consequences on the evaluation of the results, mainly unequal sample size regarding demographic characteristics and incomplete participants' data. We have attempted to minimize its impact by applying appropriate statistical methods.

The improvement in all task scores in both participant groups we observed were not explained by the statistical models we used. It is thus likely that the score improvement in Visit 2 is due to past experience of the participants with the ESO study protocol. The participants may have been more relaxed and concentrated during Visit 2, which contributed to their improved performance.

It is important to note that no specific scale was applied to measure self-disturbances and our proposed PANSS-derived Self-cluster as a measure of self disturbances has not been validated in other studies. Our findings of a relation between this subset of positive symptoms and behavioral SELF-score provide a first validation of the usefulness of this measure capitalizing on the Schneiderian first rank symptoms that presuppose self-disturbances in their definition.

The number of blocks for each condition was not high (i.e., 12 blocks of the Self-condition in the SoA task and 12 blocks in the Color task). Although this may seem low, it must be understood these were not individual events, but blocks consisting of continuous sampling of many time points that together compose the aggregate measures. While adding more blocks would increase the signal to noise ratio of our estimates and reduce the probability of false negative findings, the presented number of blocks was chosen as a tradeoff—as the experiment length poses a burden especially for the clinical participants.

The participants were not specifically tested for color blindness, which would impact the performance in the color task. However, the participants practiced the task in a training session, and only data of those, who were able to perform the task (i.e., without red-green color blindness), were recorded during the main experiment.

## Conclusions

We identified a decreased ability to attribute correctly self-produced actions in first psychotic episode schizophrenia patients, which was linked to the severity of symptoms closely related to self-disturbances and disorganized symptoms. The longitudinal analysis suggests that SoA impairment is—at least partially—associated with the intensity of respective symptoms in a state-dependent manner. Taken together, in our study, the SoA alterations in schizophrenia patients are linked to self-disturbances, which might suggest self-monitoring impairment, and to a deficit of higher cognitive abilities related to cognitive flexibility and information updating.

## Data Availability Statement

The raw data supporting the conclusions of this article will be made available by the authors, without undue reservation.

## Ethics Statement

The studies involving human participants were reviewed and approved by National Institute of Mental Health, Czechia. The patients/participants provided their written informed consent to participate in this study.

## Author Contributions

EK wrote the first draft and its subsequent versions. EB and OH performed the data analyses. PK provided the clinical assessments. OB and YZ critically revised the article. FŠ designed the study and wrote the protocol. All the authors participated in the data interpretation, commented on the draft and approved the final version of the manuscript.

## Conflict of Interest

The authors declare that the research was conducted in the absence of any commercial or financial relationships that could be construed as a potential conflict of interest.
