# Supplementary material for: Disrupted Sense of Agency as a State Marker of First-Episode Schizophrenia: A Large-Scale Follow-Up Study
Source: Front Psychiatry. 2020 Dec 18;11:570570. doi: 10.3389/fpsyt.2020.570570 (PMC7775529; doi:10.3389/fpsyt.2020.570570)
Supplement: Supplementary file 1 [file Data_Sheet_1.docx]

Supplementary Material

**Table S1 - Number of participants in all tasks and both visits**

|  | **PATIENTS** | | | |  | **CONTROLS** | | | |
| --- | --- | --- | --- | --- | --- | --- | --- | --- | --- |
|  | **SELF total** | **SELF not OK** | **SELF OK** | **SELF OK + COLOR** |  | **SELF total** | **SELF not OK** | **SELF OK** | **SELF OK + COLOR** |
| **Visit 1** | 210 | 93 | 117 | 48 |  | 147 | 30 | 117 | 33 |
| **overlap** | 100 | 12 | 43 | 10 |  | 86 | 11 | 61 | 7 |
| **Visit 2** | 136 | 49 | 87 | 44 |  | 120 | 22 | 98 | 39 |
| **Total subjects** | 246 |  | **161** | 82 |  | 181 |  | **154** | 65 |
| Numbers of participants in individual assessments. For patients, Visit 1 - time of admission to a psychiatric hospital; Visit 2 - follow-up. For controls, Visit 1 - baseline, first assessments; Visit 2 - follow-up. SELF total- number of participants who performed the SoA task; SELF (not) OK - number of participants that were (not) able to follow the instructions of the SoA task; SELF OK + COLOR- number of participants who have the control task and SELF OK. Overlap- number of participants included in both Visit 1 and V2. Total number of subjects is calculated as V1 + V1 - overlap. | | | | | | | | | |

# S1 SoA task description


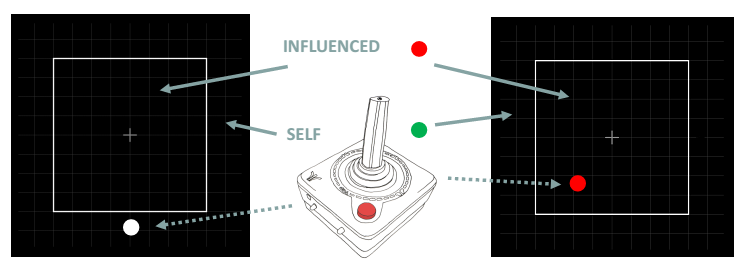
The task environment is defined by a grid with a square splitting the whole area into a peripheral and a central area (see Supplementary Figure S1a). In both SoA and control Color task, participants control a cursor by moving a joystick (Fig. S1a). Because the testing was done in an MRI scanner, participants were using a fMRI-compatible joystick placed in their dominant hand while they were watching a screen above their head reflected in mirror glasses. To be sure that the participants understand the tasks assignments, they tried both tasks using a laptop with a touchscreen connected to a monitor prior to testing itself.

**Figure S1a - Task environment:** In the SoA task (left), the cursor’s color is constant and the correct area of response depends on the presence of movement distortion at a given time point. In the Color task (right), the cursor movement is undistorted and the target area is indicated by changes of the cursor color.

The distortions in the SoA task are made by vector changes in angle (angular distortion) and distance (radius distortion) from the centre of the field (Fig. S1b). The Other- condition is initiated by moving the cursor inside the square (decreased radius during the first 2 seconds of the block), because it is more difficult to control the cursor under the influence of distortions. This manipulation suppresses false negative results in the Other- condition - e.g. participants recognize that the movement is influenced, but are not able to move swiftly into the right area. In the control Color task, the color of the cursor changes in intervals lasting between 2 to 5 seconds.


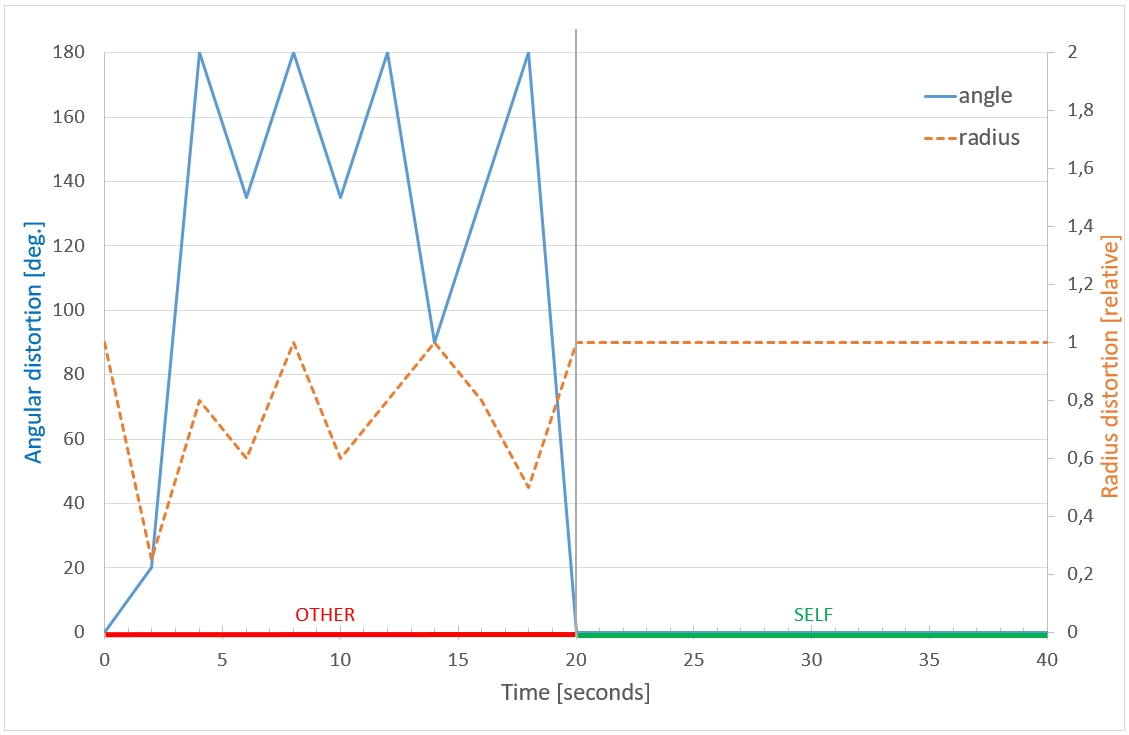


**Figure S1b** - **Graph of distortions**: The distortion introduced during the blocks of the SoA task: the angular component (solid blue) and radius component (dashed orange) joystick distortion during the influenced (OTHER, first 20s) and SELF (last 20s) conditions.


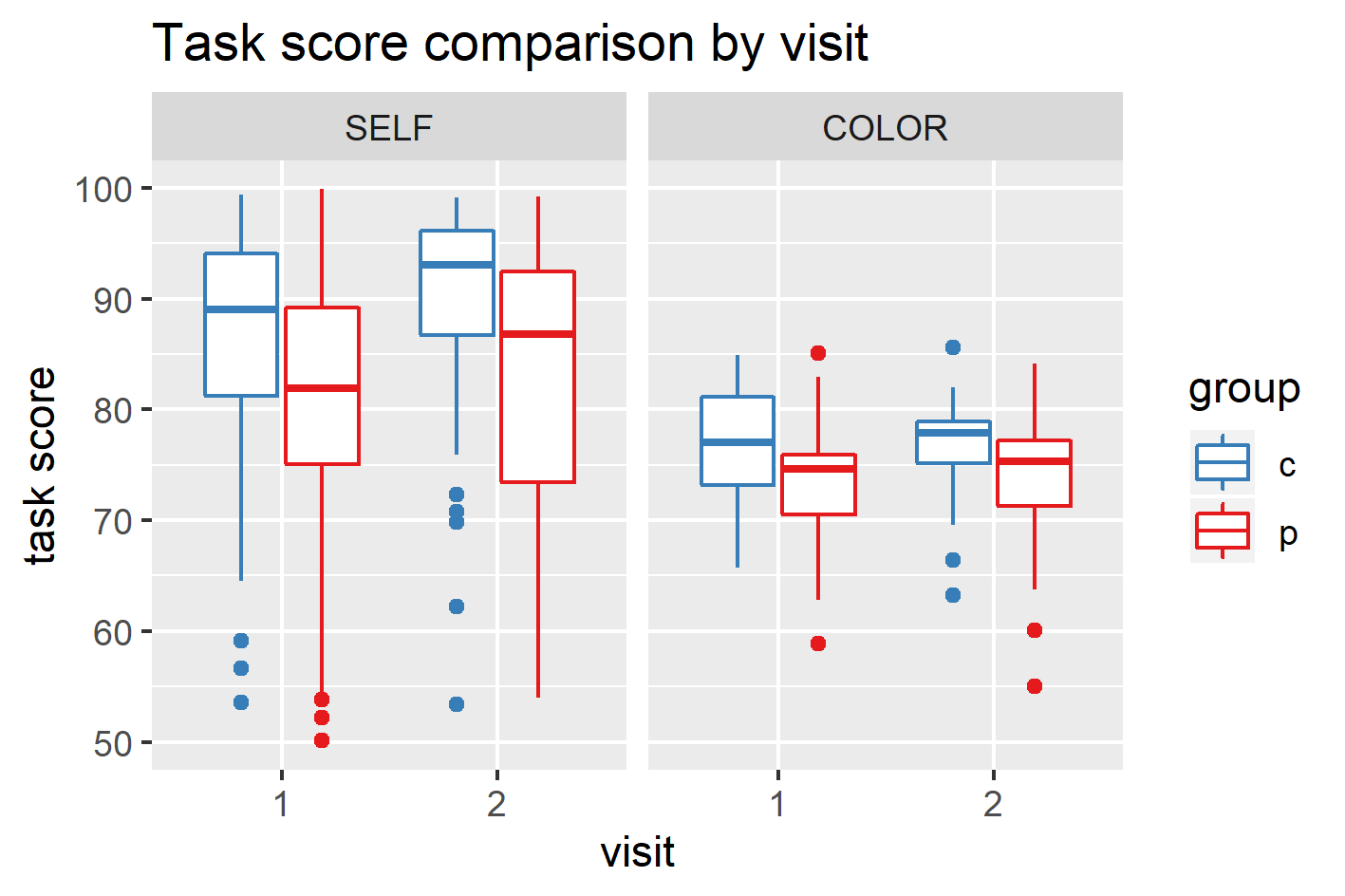


**Figure S2** - **Task score by visit**: SELF: SELF-score, COLOR: COLOR-score; c: healthy controls, p: patients

# S2 Criteria for performance evaluation and identification of non-following subjects

Only those participants who were actually performing the task according to instructions were included into the final analysis. To identify the non-conforming participants, we used the following fixed algorithm: we first converted each joystick trajectory (a series of 12 000 x,y values) by computing the Chebyshev distance (the greater of the horizontal and vertical direction) from the field center:

joyAbs(i) = max(|x(i)-cx| , |y(i)-cy|),

where *x(i),y(i)* are the horizontal and vertical coordinates of the cursor location at time *i* and *cx, cy* are coordinates of the field center. By comparing the distance value of the boundary between the inner and outer area, this metric can be used to efficiently assess in which area the cursor was at a given time point.

We computed the following two measures for each tuple of subsequent Other- and Self- blocks: i) Pearson’s correlation between the series and design matrix (0 for Other and 1 for Self blocks), and ii) ratio between the mean distance from the center during the Other and the mean distance during the Other block. From each of these values (12 for each measure, as there were 12 block pairs), we took the 25th percentile.

To identify non-following subjects, we used an additional group of 40 healthy volunteers, who were completely unfamiliar with the task and had no other instructions than to move the joystick randomly. If the participant exceeded the 95th percentile value from the distribution of the uninformed group in any of the two metrics, he/she was identified as non-following.

# S3 Modelling the group effects in the self and control tasks using mixed-effects models

The following formulae in the lme4 R package were used to model the dependency between the self and control tasks and the group effects

**model A)** self score proportional to the control task, no between-group difference

SELF.score ~ COLOR.score + age.at.V1 + sex + Visit + (1 | ID.subject)

**model B)** model A extended with different intercept for each group

SELF.score ~ COLOR.score + group + age.at.V1 + sex + Visit + (1 | ID.subject)

**model C)** model B extended with different slope in each group

SELF.score ~ COLOR.score + group + COLOR.score:group + age.at.V1 + sex + Visit + (1 | ID.subject)

**model D)** model C extended with different step change between V1 and V2 for each group and learning effect (isSecond - subset of V2 measurements, where the participants solved the task for the second time)

SELF.score ~ COLOR.score + group + COLOR.score:group + age.at.V1 + sex + Visit : grp + isSecond + (1 | ID.subject)

The formulae for models in the post-hoc evaluation was:

**models AP) and AC)** equivalent to model A, evaluated only on data of Patients’ (AP) or healthy controls (AC)

SELF.score ~ COLOR.score + age.at.V1 + sex + Visit + (1 | ID.subject)

The summary of the model comparison using likelihood ratio testing (ANOVA) is presented in Table S2, while the estimated model coefficient values are to be found in Table S3.

**Table S2:** Results of the likelihood test of models of between-group differences between the SoA and Color tasks.

|  | **Df** | **AIC** | **BIC** | **logLik** | **deviance** | **Chisq** | **Chi Df** | **Pr(>Chisq)** |
| --- | --- | --- | --- | --- | --- | --- | --- | --- |
| model A | 7 | 1249.3 | 1271.0 | -617.6 | 1235.3 | NA | NA | NA |
| model B | 8 | 1242.3 | 1267.1 | -613.2 | 1226.3 | 8.97 | 1 | 0.003 |
| **model C** | **9** | **1239.5** | **1267.4** | **-610.8** | **1221.5** | **4.78** | **1** | **0.029** |
| model D | 11 | 1242.6 | 1276.7 | -610.3 | 1220.6 | 0.91 | 2 | 0.635 |

**Table S3:** coefficients and properties of the testing between-group differences. The SELF-score was the response variable in all models.

|  | **model A** | | | **model B** | | | **model C** | | | **model D** | | | |
| --- | --- | --- | --- | --- | --- | --- | --- | --- | --- | --- | --- | --- | --- |
| *Predictors* | *β* | *CI* | *p* | *β* | *CI* | *p* | *β* | *CI* | *p* | *β* | | *CI* | *p* |
| (Intercept) | 34.36 | 10.13 – 58.59 | **0.005** | 48.62 | 23.31 – 73.93 | **<0.001** | 14.05 | -25.55 – 53.65 | 0.487 | 14.42 | | -25.36 – 54.21 | 0.477 |
| COLOR.score | 0.53 | 0.23 – 0.82 | **<0.001** | 0.39 | 0.09 – 0.69 | **0.011** | 0.84 | 0.34 – 1.34 | **0.001** | 0.83 | | 0.33 – 1.33 | **0.001** |
| age at V1 | 0.24 | 0.00 – 0.48 | **0.049** | 0.22 | -0.01 – 0.45 | 0.066 | 0.21 | -0.02 – 0.44 | 0.073 | 0.21 | | -0.02 – 0.44 | 0.072 |
| sex [f] | 2.82 | -0.63 – 6.27 | 0.109 | 1.90 | -1.50 – 5.30 | 0.273 | 2.20 | -1.16 – 5.56 | 0.200 | 2.55 | | -0.86 – 5.97 | 0.143 |
| Visit [2] | 3.25 | 0.35 – 6.14 | **0.028** | 3.15 | 0.32 – 5.99 | **0.029** | 3.19 | 0.39 – 5.99 | **0.026** | 3.91 | | -1.26 – 9.09 | 0.138 |
| group [p] |  |  |  | -5.47 | -8.99 – -1.94 | **0.002** | 46.21 | 0.12 – 92.29 | **0.049** | 45.51 | | -0.70 – 91.73 | 0.054 |
| COLOR.score: group [p] |  |  |  |  |  |  | -0.68 | -1.29 – -0.08 | **0.028** | -0.66 | | -1.27 – -0.05 | **0.035** |
| group [p] * Visit [2] |  |  |  |  |  |  |  |  |  | -2.53 | | -8.30 – 3.24 | 0.390 |
| isSecond |  |  |  |  |  |  |  |  |  | 1.05 | | -3.37 – 5.46 | 0.642 |
| **Random Effects** | | | | | | | | | | | | | |
| σ2 | 45.93 | | | 45.44 | | | 44.69 | | | | 47.50 | | |
| τ00 | 68.54 ID | | | 62.54 ID | | | 60.08 ID | | | | 56.14 ID | | |
| ICC | 0.60 | | | 0.58 | | | 0.57 | | | | 0.54 | | |
| N | 147 ID | | | 147 ID | | | 147 ID | | | | 147 ID | | |
| Observations | 164 | | | 164 | | | 164 | | | | 164 | | |
| Marginal R2 / Conditional R2 | 0.119 / 0.646 | | | 0.174 / 0.652 | | | 0.199 / 0.658 | | | | 0.202 / 0.634 | | |

# S4 Signal Detection Theory analysis

Both the SoA and the control tasks are discrimination/detection tasks and as such are suitable for analysis from the perspective of the Signal Detection Theory (SDT, Green and Swets 1966). SDT can “separate” accuracy (percentage of correct responses) into two components: sensitivity (*d’*, the ability to correctly detect the presence of some signal or discriminate two kinds of signals) and bias (*c*, the general tendency to favor one response over the other). In our case, we can try to distinguish whether the lower accuracy (SELF-score) in schizophrenia patients is due to lower sensitivity (inability to discriminate whether the cursor is under their control or not) or some response bias (i.e. to stay more often in the inner or outer corridor of the square regardless of the condition, which can partially reflect the tendency to under- or over-attribute control over the cursor, but there may be other reasons for staying more often in one of the corridors). Both of these measures are in standard units (z-scores). We further divided c by d’ to obtain a relative measure of bias, *c’*, because in a task of different discrimination difficulty a different c is needed to obtain the same amount of bias (Macmillan and Creelman 2004). Both d’ and c’ have the same scale in both the SoA and control tasks and thus can be compared between these two tasks. For the bias, positive values mean tendency to stay in the inner corridor, i.e., bias towards attributing motion to the Other in the SoA task.

The SDT measures need to be computed from both the Self and Other conditions. In the main analysis, we included only performance in the Self blocks, because in the Other blocks we cannot safely distinguish whether participants moved within a given section of the square because of their choice (judgement of who is in control of the cursor) or because of the cursor distortions and this would include noise into our analysis. Moreover, the Other blocks started with an automatic drift towards the centre of the square, i.e., towards the “correct response”, which could inflate the measured sensitivity of participants to discriminate the actual authorship of movements. This drift was present in both groups and group comparisons should subtract the influence of automatic drift on both measures and leave only difference due to factors of interest: sensitivity to authorship, under- or over-attribution and influence of sensorimotor abilities, which should be further subtracted to some degree by including the control task in the analysis. Nevertheless, the results of these SDT analyses should be interpreted with caution.

In two separate linear mixed-effects models we modelled d’ and c’ as dependent variables using the lme4 R package and the following formulae:

d’ ~ isPatientGroup * isSoATask + isSecond + isMale * ageAtV1Scaled + (1 + isColorTask | participantID)

c’ ~ isPatientGroup * isSoATask + isSecond + isMale + ageAtV1Scaled + (1 + isColorTask | participantID)

This model structure was chosen based on theoretical and statistical (to minimize AIC, the Akaike Information Criterion) reasons: We wanted to test whether patients have specifically different performance in the SoA than the Color task than the controls do (interaction isPatientGroup:isSoATask). The indicator variable isSecond was set to 1 for the second experience with the tasks and as a mere additive term reflects experience with the tasks as well as general effects of time. For patients, additionally, this variable is strongly correlated with the clinical state (symptoms, because of medication and therapy) and could obfuscate the interaction between group and task if added as an interaction with group and task. Also, models with a mere additive term had lower AIC (Akaike Information Criterion). Demographics (sex and age at first visit) were added as additively, and for d’ with a surprising interaction between them, again to minimize AIC. The random-effects structure was also chosen to obtain final minimal AIC. The results are summarized in Table S4.

**Table S4:** Table shows estimates and 95% confidence intervals (CI) for the regression coefficients and p-values computed using degrees of freedom obtained via the Satterthwaite's method (R package lmerTest).

|  | **model of d’** | | | **model of c’** | | |
| --- | --- | --- | --- | --- | --- | --- |
| *Predictors* | *β* | *CI* | *p* | *β* | *CI* | *p* |
| (Intercept) | 1.92 | 1.63 – 2.20 | **<0.001** | 0.06 | -0.06 – 0.17 | 0.339 |
| isSoATask | 0.81 | 0.68 – 0.94 | **<0.001** | -0.04 | -0.11 – 0.03 | 0.248 |
| isPatientGroup | -0.18 | -0.28 – -0.08 | **<0.001** | 0.07 | 0.01 – 0.13 | **0.031** |
| isSecond | 0.03 | -0.05 – 0.12 | 0.443 | -0.05 | -0.08 – -0.01 | **0.006** |
| isMale | -0.39 | -0.77 – -0.01 | **0.048** | 0.03 | -0.02 – 0.08 | 0.240 |
| ageAtV1 Scaled | -0.02 | -0.03 – -0.01 | **<0.001** | -0.00 | -0.01 – 0.00 | 0.118 |
| isSoATask:isPatientGroup | -0.28 | -0.45 – -0.10 | **0.003** | -0.04 | -0.13 – 0.06 | 0.444 |
| isMale:ageAtV1 Scaled | 0.02 | 0.00 – 0.03 | **0.010** |  |  |  |
| **Random effects** |  |  |  |  |  |  |
| σ2 | 0.09 |  |  | 0.01 |  |  |
| τ00 | 0.01 ID |  |  | 0.03 ID |  |  |
| τ11 | 0.13 ID.isSoATask | | | 0.07 ID.isSoATask | | |
| ρ01 | 1.00 ID |  |  | -0.49 ID |  |  |
| ICC | 0.08 ID |  |  | 0.76 ID |  |  |
| Observations | 328 |  |  | 328 |  |  |
| Marginal R^2^ / Conditional R^2^ | 0.431 / 0.736 | | | 0.062 / 0.838 | | |

The ability to discriminate the relevant signals (motion under own control vs. with external influence or red vs. green color) (d’) was generally higher in the SoA task than in the Color task. It was also generally lower for patients than for controls across both tasks. Importantly, a significant interaction between task and group showed that this ability was even lower specifically for patients in the SoA task. That means, patients had a specific impairment in the ability to tell whether the movement of the cursor was under their control, compared to control participants and compared to a control task, which controls for general sensorimotor abilities and perceptual and attentional capacities. Interestingly, this ability was lower for males of youngest age, but while it was constant for males, it decreased with age but only for women (Figure S4).


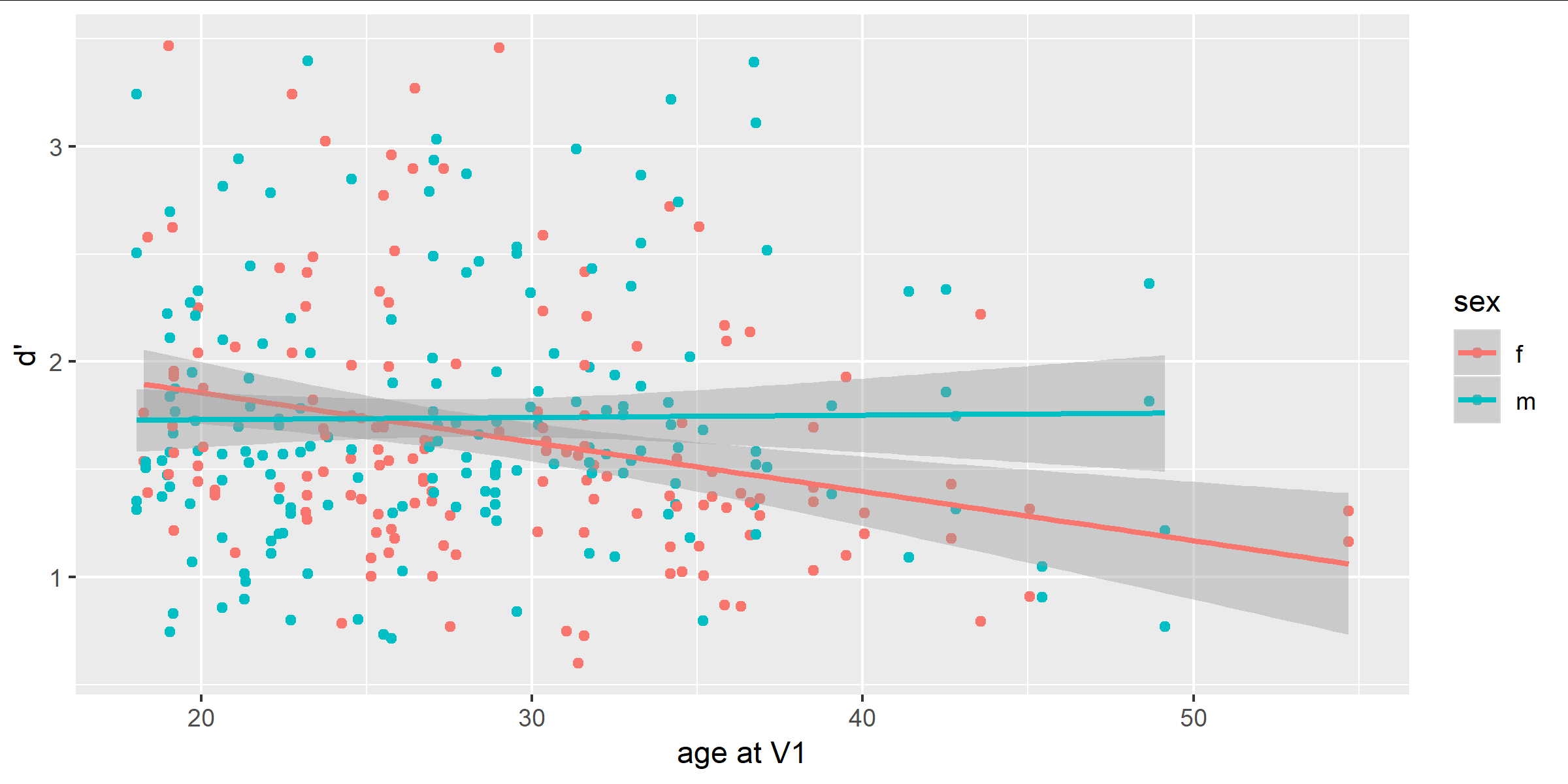


**Figure S4**: d’ as a function of age at visit 1 and sex (f: female, m: male), with best fit lines and shaded 95% CI.

Patients had a significantly more positive bias (c’) than the controls, i.e., they tended to keep in the inner area more often than healthy controls (who in general had a somewhat negative bias, i.e. kept more often in the outer region). However, there was no effect nor interaction with a task, so it cannot be said the bias was specific to self-attribution (or due to automatic drifts towards the inner region in the SoA task), but rather to the motion of the cursor with the joystick in general. Experience with the task made the bias significantly more negative, i.e., participants tended to keep in the outer area of the square more if they did the tasks for the second time (Figure S5). Overall, these results suggest that the impaired accuracy in the SoA task in patients was to a larger degree due to a lower sensitivity than response bias.


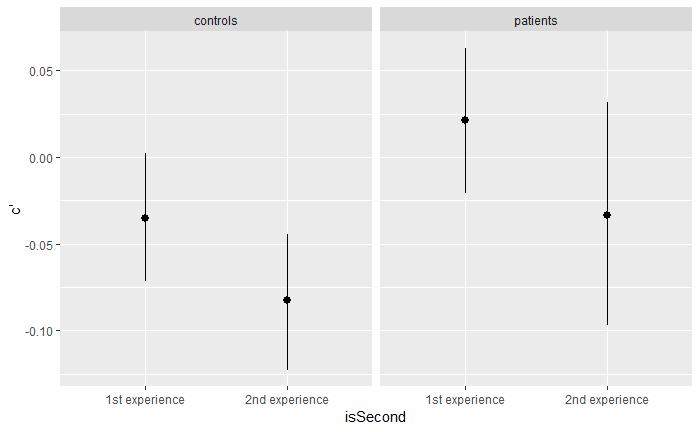


**Figure S5:** Mean c’ and bootstrapped 95% CI per group and experience with task, collapsed over tasks.

# S5 Evaluation of symptom severity vs task scores

The evaluated linear mixed-effects model was of the following form:

SELF-score ~ PANSS-Score + Visit + isSecond + (1|ID)

where the PANSS.Score was replaced by the respective PANSS factor/subscale and SELF-score alternatively by COLOR-score, according to Table S5 below. Visit models score differences in the score of interest, isSecond models the learning effect for subjects, who solved the task for the second time during the second visit. Significance of the was evaluated using the Satterthwaite's method.

**Table S5: Dependence of SELF and COLOR scores on PANSS subscales**

| **Task** | **PANSS subscale** | **beta** | **t value** | ***p*** | ***p_corr_*_,_ n=6** |
| --- | --- | --- | --- | --- | --- |
| **SELF** | **Self-cluster** | **-1.02** | **-2.79** | **0.006** | **0.029** |
|  | Positive Factor | -0.58 | -2.06 | 0.041 | 0.164 |
|  | Negative Factor | -0.28 | -1.54 | 0.126 | 0.379 |
|  | **Disorganized Factor** | **-1.31** | **-3.02** | **0.003** | **0.017** |
|  | Excited Factor | 0.26 | 0.49 | 0.624 | 0.741 |
|  | Depressed Factor | 0.37 | 0.90 | 0.371 | 0.741 |
| **COLOR** | Self-cluster | -0.67 | -1.46 | 0.147 | 0.588 |
|  | Positive Factor | -0.36 | -1.13 | 0.264 | 0.791 |
|  | Negative Factor | -0.37 | -1.82 | 0.073 | 0.364 |
|  | Disorganized Factor | -0.31 | -0.64 | 0.521 | 1.000 |
|  | Excited Factor | 0.38 | 0.54 | 0.590 | 1.000 |
|  | Depressed Factor | 0.99 | 2.41 | 0.019 | 0.113 |
| SELF: SELF-score in SoA task, COLOR: score in control Color task (left column); PANSS subscales: positive, global, negative, total, Self-cluster: custom subscale, PosFact: Positive Factor in Wallwork's model. Results using linear mixed-effects model,  on 197 observations from 159 subjects. Model formula SELForCOLOR ~ PANSS.subscore + VISIT + isSecond + (1 \| ID)  Corrected *p*-values were computed using the Holm method (N=6) | | | | | |

**References**

Green, David Marvin, and John A. Swets. 1966. *Signal Detection Theory and Psychophysics*. Vol. 1. Wiley New York.

Macmillan, Neil A., and C. Douglas Creelman. 2004. *Detection Theory: A User’s Guide*. Psychology press.
